# Equivalence of Self- and Staff-Collected Nasal Swabs for the Detection of Viral Respiratory Pathogens

**DOI:** 10.1371/journal.pone.0048508

**Published:** 2012-11-14

**Authors:** Manas K. Akmatov, Anja Gatzemeier, Klaus Schughart, Frank Pessler

**Affiliations:** 1 Department of Epidemiology, Helmholtz Centre for Infection Research, Braunschweig, Germany; 2 Department of Infection Genetics, Helmholtz Centre for Infection Research, Braunschweig, Germany; 3 University of Veterinary Medicine Hannover, Hannover, Germany; 4 Division of Rheumatology and Immunology, University Children’s Hospital, Technical University Dresden, Dresden, Germany; Royal Melbourne Hospital, Australia

## Abstract

**Background:**

The need for the timely collection of diagnostic biosamples during symptomatic episodes represents a major obstacle to large-scale studies on acute respiratory infection (ARI) epidemiology. This may be circumvented by having the participants collect their own nasal swabs. We compared self- and staff-collected swabs in terms of swabbing quality and detection of viral respiratory pathogens.

**Methodology/Principal Findings:**

We conducted a prospective study among employees of our institution during the ARI season 2010/2011 (December-March). Weekly emails were sent to the participants (n = 84), reminding them to come to the study center in case of new symptoms. The participants self-collected an anterior nasal swab from one nostril, and trained study personnel collected one from the other nostril. The participants self-collected another two swabs (one from each nostril) on a subsequent day. Human β-actin DNA concentration was determined in the swabs as a quality control. Viral respiratory pathogens were detected by multiplex RT-PCR (Seeplex RV15 kit, Seegene, Eschborn, Germany). Of 84 participants, 56 (67%) reported at least one ARI episode, 18 participants two, and one participant three. Self-swabbing was highly accepted by the participants. The amount of β-actin DNA per swab was higher in the self- than in the staff-collected swabs (p = 0.008). β-actin concentration was lower in the self-swabs collected on day 1 than in those collected on a subsequent day (p<0.0001). A respiratory viral pathogen was detected in 31% (23/75) of staff- and in 35% (26/75) of self-collected swabs (p = 0.36). With both approaches, the most frequently identified pathogens were human rhinoviruses A/B/C (12/75 swabs, 16%) and human coronavirus OC43 (4/75 swabs, 5%). There was almost perfect agreement between self- and staff-collected swabs in terms of pathogen detection (agreement = 93%, kappa = 0.85, p<0.0001).

**Conclusions/Significance:**

Nasal self-swabbing for identification of viral ARI pathogens proved to be equivalent to staff-swabbing in this population in terms of acceptance and pathogen detection.

## Introduction

The need for the timely collection of diagnostic biosamples (such as nasal swabs) during acute symptomatic episodes represents a major obstacle to large-scale studies on acute respiratory infection (ARI) epidemiology. This may be circumvented by having the participants collect their own nasal swabs (“self-collected nasal swabs” or “self-swabbing”). This method has been shown to be highly acceptable and feasible in various populations, e.g. among parents who collected nasal swabs from their children [1] or adults who collected swabs from themselves [2] (also reviewed in [3]). While the mere feasibility of nasal self-swabbing has thus been amply demonstrated, efforts to validate the diagnostic equivalence of self-swabbing compared to staff-swabbing are still ongoing. In a sample of 38 individuals with ARI symptoms, Larios et al. compared self-collected midturbinate swabs with staff-collected nasopharyngeal swabs (gold standard) that were collected the same day. The self-collected swabs had a sensitivity of 86% for the detection of respiratory pathogens compared to the gold standard [4]. Luinstra et al. found similar detection rates for respiratory pathogens between self- and staff-collected midturbinate swabs when one staff-collected and one self-collected swab were taken from opposite nostrils during the same visit to a campus health center [5]. Ip et al. investigated the validity of self-collected nasal (posterior nares) and pharyngeal swabs to detect influenza virus infection and came to the conclusion that self-swabs may be a good alternative [6]. While these results do provide substantial evidence for the validity of self-collected midturbinate swabs, anterior nasal swabs have not been evaluated in this respect. Moreover, self-collected nasal swabs collected on separate days have not been compared with each other.

In the present study, we thus compared quality of swabbing and efficiency of viral detection of anterior nasal swabs in the following scenarios: 1) self- vs. staff-collected swabs that were taken during the same visit to the study center (day 1), and 2) self- and staff-collected swabs from day 1vs. self-collected swabs that were obtained at home on a later day.

## Materials and Methods

### Sample and Study Design

A prospective study was conducted during the 2010/2011 ARI season among a convenience sample of employees of our institution, the Helmholtz Centre for Infection Research in Braunschweig, Germany. In December 2010, employees (18 to 69 years old) were sent messages through the internal e-mail system inviting them to participate in the study. This invitation contained a link to the institutional intranet where information about the study was made available in English and German. Individuals planning to leave Braunschweig during the study period and staff members of the Departments of Epidemiology and Infection Genetics were not eligible to participate, the latter due to concern over a potential conflict of interest.

At the baseline visit (December 2010–January 2011), the study aims were explained to the participants and informed consent was obtained. A self-administered questionnaire was used to collect basic sociodemographic data. At the end of the ARI season (April/May 2011) the study participants completed a short acceptance questionnaire. All participants received a remuneration of 5 €.

### Ethics Statement

The study was approved by the Ethics Committee of the State Board of Physicians of the German Federal State of Lower Saxony.

### Early Detection of ARI Symptoms and Collection of Nasal Swabs

**Figure 1:**
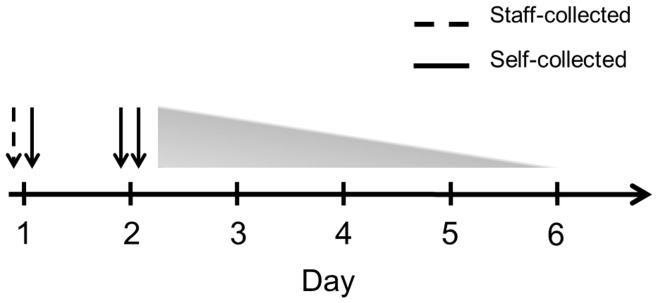
Timeline of staff-collected (interrupted line) and self-collected (solid line) swabs. A staff-collected and a self-collected swab were obtained on day 1 from separate nostrils. The participants were instructed to collect a self-swab from each nostril the next day, but the actual day of self-swabbing ranged from day 2 to day 6, as indicated by the triangle.

During January–March 2011 weekly e-mail messages were sent to the participants reminding them to come to the study center within 7 days of onset of at least one of the following symptoms: sudden onset of stuffy or runny nose, cough, sore throat, headache, malaise, chills, or fever, defined as body temperature >38°C. In the study center, a trained staff member (A.G.) obtained a nasal swab (regular flocked swab, Copan, Brescia, Italy, product number 359C) from the participant’s left nostril and instructed him/her how to perform a self-swab. The participants also received written and visual instructions for nasal self-swabbing. The participants then self-collected a swab from the right nostril. Briefly, the swab was to be inserted into the nostril to the point where the basal edge of the flocked tip had just entered the nostril, corresponding to a depth of insertion of approx. 1 cm. The swab was then rotated three times, being careful to swab all 360° of the anterior nasal lining and to include the superior recess (“Little’s area”) and the latero-inferior recess. The swab was then placed into 1 ml universal viral transport medium (Copan). A swabbing kit containing written and visual self-swabbing instructions, two nasal swabs and two vials of 1 ml transport medium was then given to the participants with the request to self-collect two nasal swabs (one from each nostril) at home the next day, to place each swab in 1 ml transport medium and to return the swabs to the study center as soon as possible. The swabs were stored at −70°C until analysis. The timeline of the study is shown in Fig. 1.

**Table 1:** Acceptability of nasal swabbing.

Items	Median (IQR)
The collection of the nasal swab by the study personnel was acceptable (n = 56)*	5 (5–5)
Collecting the nasal swab myself was acceptable (n = 56)*	5 (5–5)
I felt comfortable when the study personnel collected the swab (n = 56)*	5 (4–5)
I felt comfortable when taking the swab myself (n = 56)*	5 (5–5)
I would prefer taking a nasal swab myself and not having it taken by study personnel (n = 56)*	3 (2–3)
Nasal self-swabbing was easy to perform (n = 56)*	5 (5–5)
The instructions how to take the self-swab were understandable (n = 56)*	5 (5–5)
I would participate in a study where nasal swabs are to be taken by study personnel (n = 77)**	5 (5–5)
I would participate in a study where nasal swabs are to be taken by myself (n = 76)**	5 (5–5)

* only those participants who collected at least one nasal swab.

** all participants.

### Laboratory Analysis

Detection of human β-actin coding sequences was used as a measure of sample adequacy, assuming that the amount of β-actin gene DNA can serve as proxy for the presence of human epithelial cells [7], [8]. DNA was extracted from a 350 µl aliquot of transport medium with the AllPrep DNA/RNA Micro Kit (Qiagen GmbH, Hilden, Germany). β-actin DNA quantification was tested by real-time PCR with QuantiTect SYBRGreen PCR (Qiagen GmbH, Hilden, Germany) using the primers 5′ CCA ACC GCG AGA AGA TGA CC 3′ (forward) and 5′ GAT CTT CAT GAG GTA GTC AGT 3′ (reverse), corresponding to positions (5′) 382–617 (3′) of the human β-actin gene (Eurofins mwg Operon, Ebersberg, Germany). This resulted in amplification of a 236 bp fragment, possessing a molecular weight of 15.8 kD based on an assumed average molecular weight of 660 Dalton per basepair. Serial dilutions of the plasmid eTC GFP β-actin ΔZip (Plasmid 27124 by Addgene, 1 Kendall Sq. Ste. B7102 Cambridge, MA 02139, USA), which contains these β-actin gene sequences in a pcDNA3.1 backbone, were analyzed in parallel to obtain the standard curve. β-actin DNA concentration was determined in all four swabs. The values of the two swabs that were self-collected at home were pooled. The technician performing the laboratory analyses was not blinded as to whether a swab was staff- or self-collected.

#### Detection of viral respiratory pathogens

RNA was extracted from 200 µl aliquots of transport medium (UTM Kit, Copan, Brescia, Italy) with the QIAamp MinElute Virus Spin Kit (Qiagen GmbH, Hilden, Germany). cDNA was synthesized with the Transcriptor First Strand cDNA Synthesis Kit (Roche Diagnostics GmbH, Mannheim, Germany) and tested by multiplex PCR (Seeplex RV15 ACE Detection kit, Seegene Germany, Eschborn, Germany) for the presence of any of 15 human viral respiratory pathogens (adenovirus A/B/C/D/E, human metapneumovirus, enterovirus, bocavirus 1/2/3/4, human coronavirus 229E/NL63 and OC43, parainfluenza virus 1, 2, 3 and 4, influenza virus A and B, respiratory syncytial virus A and B, and rhinovirus A/B/C), following the manufacturer’s recommendations except that 4 µl instead of 1 µl cDNA was used as input for the PCR reaction.

**Figure 2:**
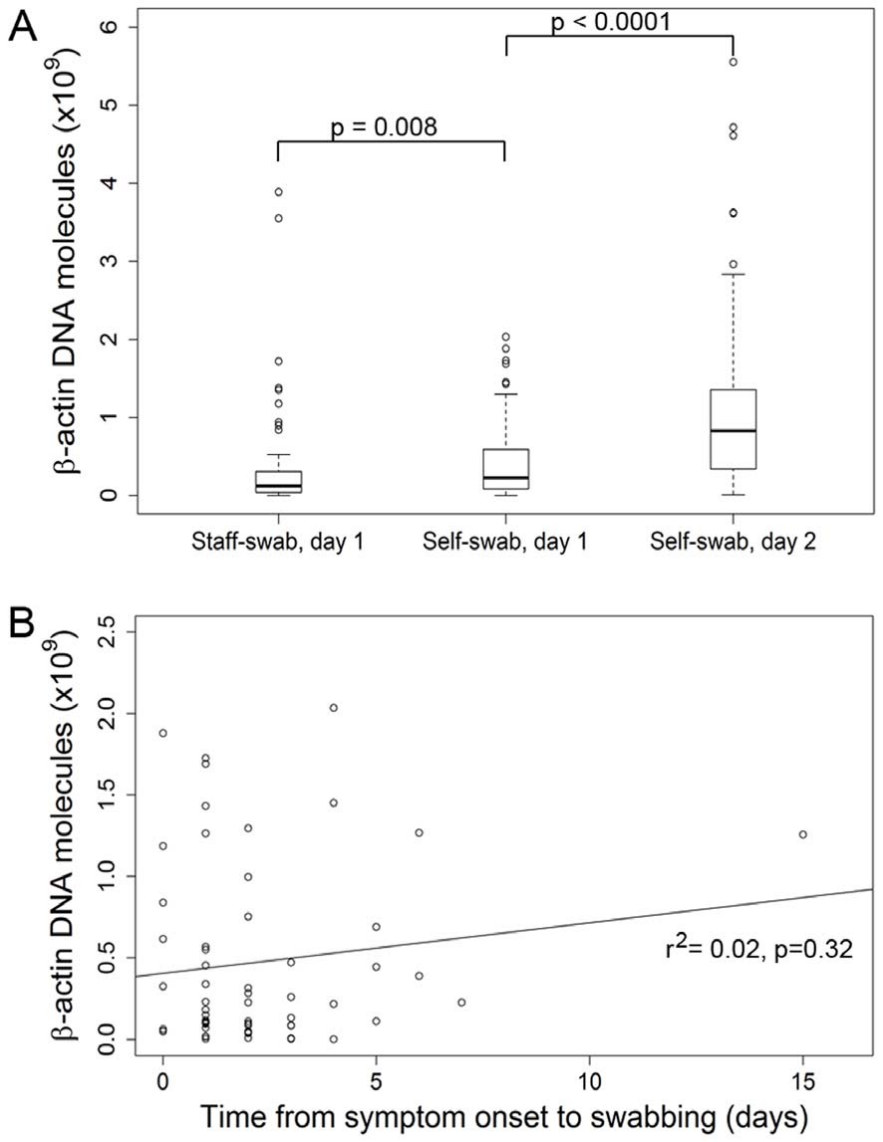
Human β-actin DNA concentration in staff- and self-collected swabs. A. Human β-actin DNA concentration in staff- and self-collected swabs obtained according to the time scheme shown in Fig. 1. β-actin DNA concentration was determined by real-time PCR and is plotted on the y-axis as the number of molecules per swab. Boxes: upper border, 75^th^ percentile; lower border, 25^th^ percentile; bold horizontal line, median; whiskers, minimum and maximum excluding outliers (circles); circles, outlying values exceeding the 75^th^ percentile by >1.5 times the height of the box. B. β-actin DNA concentration per swab in relation to time elapsed between onset of ARI symptoms and the day of swabbing (r^2^ = 0.02, p = 0.32).

### Acceptability

Satisfaction and acceptability were assessed using a nine-item questionnaire. Participants rated each item on a five-point Likert scale with 1 indicating strong disagreement, 2 disagreement, 3 neither disagreement nor agreement, 4 agreement, and 5 strong agreement. Some of these items were reverse-phrased to reduce response bias.

**Table 2:** Viral respiratory pathogens detected in staff-and self-collected swabs obtained in the study center^1^.

Pathogen	%
None	64
Human rhinovirus A, B or C	16
Human coronavirus OC43	5.3
Parainfluenza virus 1, 2, 3 or 4	5.3
Respiratory syncytial virus A or B	4.0
Human coronavirus 229E/NL63	2.7
Influenza A or B	2.7
Human metapneumovirus	0
Adenovirus A/B/C/D/E	0
Enterovirus	0
Bocavirus 1/2/3/4	0

1 Values represent the percentages of pairs of staff- and self-collected swabs obtained in the study center (total n = 75 swab pairs, collected from 56 participants) in which a given pathogen was detected by real-time PCR in at least one swab.

### Statistical Analysis

Data were described by percentage for categorical variables and median with interquartile range (IQR) for continuous variables. Agreement between self- and staff-collected nasal swabs in the detection of respiratory pathogens was examined with Cohen’s kappa (κ) statistic using the following classification: <0 = poor; 0–0.2 = slight; 0.21–0.4 = fair; 0.41–0.6 = moderate; 0.61–0.8 = substantial; and 0.81–1 = nearly perfect agreement [9]. The McNemar test was used to compare proportions of positive swabs between paired samples. Log transformation did not lead to normal distribution of the β-actin DNA concentration values. Thus, the non-transformed values were used and differences between staff- and self-collected swabs were examined with the Wilcoxon Signed Ranks test. β-actin DNA concentration values were also divided into quartiles and examined for association with positivity status using a test for trend. The statistical program SPSS for Windows, version 19, was used for all analyses.

**Figure 3:**
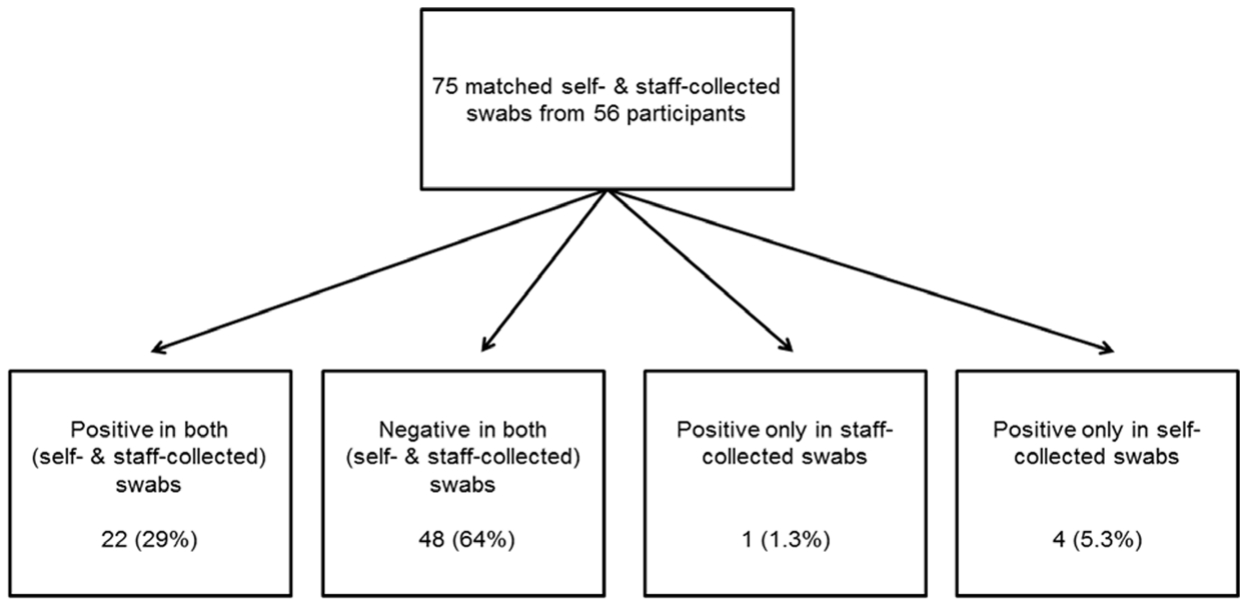
Agreement in pathogen detection between staff- and self-swabs collected on the same day. Swabs were designated positive if any of 15 respiratory viral pathogens were detected by real-time PCR (Seeplex RV15 ACE Detection kit, Seegene Germany, Eschborn, Germany). Concordant scenarios are shown on the left, discordant scenarios on the right.

## Results

Eighty-four participants responded to the invitation e-mail, corresponding to a response rate of 18%. Seventy-two participants (86%) were women, the median age was 37 years (range, 28–46). About half of the respondents had a university degree (including university of applied sciences) and about 12% were born outside Germany.

**Table 3:** Sensitivity and specificity of self-collected swabs, obtained in the study center, to detect viral respiratory pathogens (compared to staff-collected swabs)*.

Pathogens	Positive either in staff-or self-collected swabs	Sensitivity, %(95% confidence intervals)	Specificity, %(95% confidence intervals)
Human rhinovirus A/B/C	12	100 (76–100)	100 (93–100)
Human coronavirus OC43	4	100 (34–100)	96 (85–99)
Parainfluenza virus 1, 2, 3 or 4	4	100 (44–100)	98 (89–99)
Human coronavirus 229E/NL63	2	100 (34–100)	100 (93–100)
Influenza A or B	2	100 (21–100)	98 (89–99)
Respiratory syncytial virus A or B	3	67 (21–94)	100 (93–100)
**All pathogens**	**27**	**96 (79–99)**	**92 (81–97)**

* Results are not adjusted for dependence among observations; ∼25% of the paired swabs were from participants who had more than one ARI episode during the study period.

### Acceptability

Overall, both staff- and self-collected nasal swabs were highly accepted (Table 1). Most participants reported that instructions how to collect the swab were easy to understand and that the nasal self-swab was easy to perform. The participants did not make a preference regarding self- or staff-collected swabs (Table 1).

**Figure 4:**
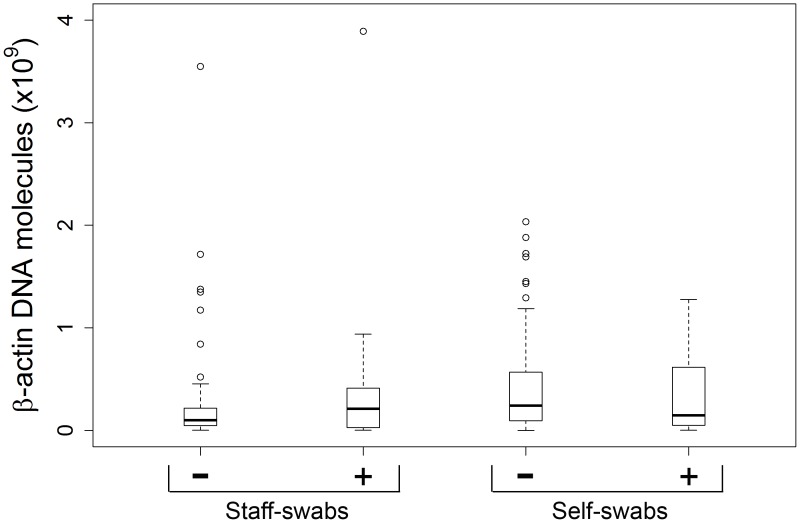
Relation between β-actin DNA concentration and viral positivity status in staff- and self-collected swabs collected on the same day. Analysis based on the data set used for Figure 2, but stratified according to viral detection status (+ positive, − negative). Y-axis: human β-actin DNA molecules per swab. P values: p = 0.186 for comparison between negative and positive staff-swabs; p = 0.404 for comparison between negative and positive self-swabs (Wilcoxon rank sum test).

### Detection of ARI Symptoms and Collection of Nasal Swabs

Fifty-six of 84 (67%) participants reported at least one ARI episode, 18 (32%) participants reported two episodes, and one participant reported three, resulting in a total of 75 ARI episodes for the final analysis. The number of ARI symptoms reported in a single ARI episode ranged from one to seven (median, 3.0). Thus, 75 matched pairs of staff- and self-collected nasal swabs, performed on the same day, were available. About 14% of these were taken on the day of symptom onset (day 1), 33% on day 2, 22% on day 3, 12% on day 4, and 19% on or after day 5. The additional set of self-collected swabs, which was to be collected at home the next day, was obtained in the majority of ARI episodes (71/75, 95%), for 67/71 (94%) of which swabs from both nostrils were returned, and from only one side in the remaining four. Sixty-two percent of these swabs were indeed collected the next day (day 2) and the latest one on day 6 (Fig. 1).

### Laboratory Results

When comparing the staff- and self-swabs collected on day 1, β-actin DNA concentration was higher in the self-collected swabs (p = 0.008) (Fig. 2A). The β-actin DNA concentration was higher in the self-swabs collected on or after day 2 than in the self-swabs from day 1 (p<0.0001). Interquartile distance was smallest in the staff-collected swabs and greatest in the swabs that were self-collected at home, indicating lower variability in the staff-collected than in the self-collected swabs. β-actin DNA levels did not correlate with the duration of symptoms preceding the day of swab collection (Fig. 2B).

A respiratory viral pathogen was detected in 31% (23/75) of staff- and in 35% (26/75) of self-collected swabs collected on day 1 (p = 0.36, McNemar test). In both, the most frequently identified pathogens were human rhinoviruses A, B or C (12/27 positive swabs, 44%), human coronavirus OC43 (4/27 swabs, 15%) and parainfluenza viruses 1, 2, 3 or 4 (4/27, 15%). Table 2 contains the complete list of detected pathogens, expressed as percentages of all 75 swab pairs collected on day 1 in which a pathogen was detected in at least one of the two swabs. There was nearly perfect agreement between the staff- and self-swabs collected on day 1 in terms of pathogen detection (percent agreement = 93%, κ = 0.85, Fig. 3). The overall sensitivity of self-collected swabs to detect a respiratory pathogen was 96%, compared to the staff-collected swabs (Table 3). This very good agreement between the staff- and self-collected swabs from day 1 is also evident in the flow diagram shown in Fig. 3.

Analyzing the results obtained with the self-swabs from day 2 or later, a viral pathogen was detected in 39% (26/67) of the swab pairs when the results of both nostrils were combined. When the swabs from each side were considered separately, a pathogen was detected in 31% (21/67) of the self-collected swab from the right and in 37% (25/67) of swabs from the left nostril. However, this apparent difference was not significant (p = 0.22, McNemar test). Comparing viral detection of these self-swabs with that of the staff-collected swabs from day 1 revealed substantial agreement (percent agreement = 85%, κ = 0.67). The detection of a viral pathogen was independent of the amount of β-actin DNA in both staff- and self-swabs collected on day 1 (Fig. 4). Likewise, there was no association between viral positivity status and β-actin DNA levels across all samples (p for trend = 0.943).

## Discussion

This prospective study comparing staff- and self-collected nasal swabs for the detection of ARI pathogens clearly demonstrated the validity of self-swabbing; specifically, self-swabbing was not inferior in terms of acceptance, satisfaction, sample adequacy, and viral detection rate. Of note, we observed excellent agreement in viral detection between self- and staff-collected swabs collected the same day. The agreement between the swabs that were self-collected at home and the staff-collected swabs was somewhat less pronounced, but still was classified as substantial (κ = 0.67). This lower agreement may be explained by the time lag between collection of the staff-swabs and the self-swabs at home, which spanned up to 6 days and resulted in five additional positive self-swabs, but only two additional negative ones. Thus, taken together, the results strongly indicate equivalence of self-collected and staff-collected swabs in this study population.

Noteworthy differences were detected in β-actin DNA concentration, which was used to quantify the amount of host cells and thus served as a measure of sample adequacy [8]. Median β-actin DNA concentration was significantly higher in the self-collected than in staff-collected swabs, but also in the swabs that were self-collected at home than in the self-collected swabs from day 1. Taken together, these results suggest (1) that the participants applied higher swabbing pressure than the trained staff member, likely due to high confidence in the self-swabbing procedure, and (2) that a training effect resulted in yet greater confidence and more vigorous swabbing when self-swabbing was repeated at home ≥1 day later. In support of this notion, in a study comparing staff- and self-collected swabs that were obtained the same day Smieja et al. observed a higher number of epithelial cells and a tendency toward a higher β-actin DNA level in a second self-swab that was collected immediately after the first one [8]. Alternatively, the presence of leukocytes in purulent secretions may have contributed to β-actin levels in some swabs. However, we do not consider this to be a major contributing factor since most participants did not have purulent secretions. Another theoretical reason for the higher β-actin DNA levels in the swabs that were self-collected at home might have been the longer disease duration, leading to higher shedding of epithelial cells or higher leukocyte numbers in the secretions. However, as shown in Fig. 2B, there was no association between duration of symptoms and β-actin concentration in the swabs, thus ruling out this possibility in the present study. Of note, more thorough sampling (as reflected by higher β-actin DNA levels) did not improve viral detection in our study, suggesting that the amount of host cells on the swabs was near the optimum in most cases. Alternatively, the sampling of host cells may not be a major determinant for the detection of ARI viruses due to the presence of sufficient amounts of viral particles in nasal secretions.

A criticism of studies comparing staff- and self-collected swabs from the same day has been that the participants might feel more confident in the presence of study personnel [10] and that self-swabs might actually be inferior when collected in the absence of study personnel. We tested this hypothesis by including two additional nasal swabs which were self-collected at home ≥1 day later. Comparison of these swabs with staff-collected swabs collected on day 1 revealed good agreement in terms of viral detection and even better sample adequacy, thus demonstrating the diagnostic equivalence of self-swabbing in an unsupervised setting, provided that the participants have been trained adequately.

### Limitations

It should be kept in mind that only two individuals with PCR-proven influenza virus infection were detected. Therefore, anterior nasal self-swabbing for the detection of influenza virus infection still needs to be validated in dedicated studies. Recently, a flocked mid-turbinate swab was developed and turned out to be superior to the gold standard nasopharyngeal swab in terms of ARI virus detection [8]. The anterior nasal swab used in the present study has not been compared to this midturbinate swab. This should be done in a future study. Our findings are also limited by the fact that the study was conducted in a selected study population recruited within a research institution. However, the population included a mixture of scientific, clerical and support staff. Notably, education level spanned a broad range and did not influence viral detection or β-actin DNA levels. Nonetheless, our findings need to be validated in future studies using random samples drawn from the general population.

Due to much lower expenses for personnel and travel, self-collection would be a highly cost-efficient way to obtain diagnostic nasal swabs in medium and large scale population-based studies on ARI epidemiology [3]. The presented study adds significantly to the growing body of evidence demonstrating its diagnostic equivalence to staff-collection.

### Conclusions

Nasal self-swabbing proved to be an acceptable, feasible and valid method to identify viral respiratory pathogens in this selected adult population. Its applicability in the general population should be tested in future studies.
